# Percutaneous valve in all four positions

**DOI:** 10.1007/s12471-022-01691-x

**Published:** 2022-04-27

**Authors:** H. M. Aarts, A. O. Kraaijeveld, P. R. Stella, M. Voskuil

**Affiliations:** Department of Cardiology, University Medical Center Utrecht, Utrecht, The Netherlands

**Fig. 1:**
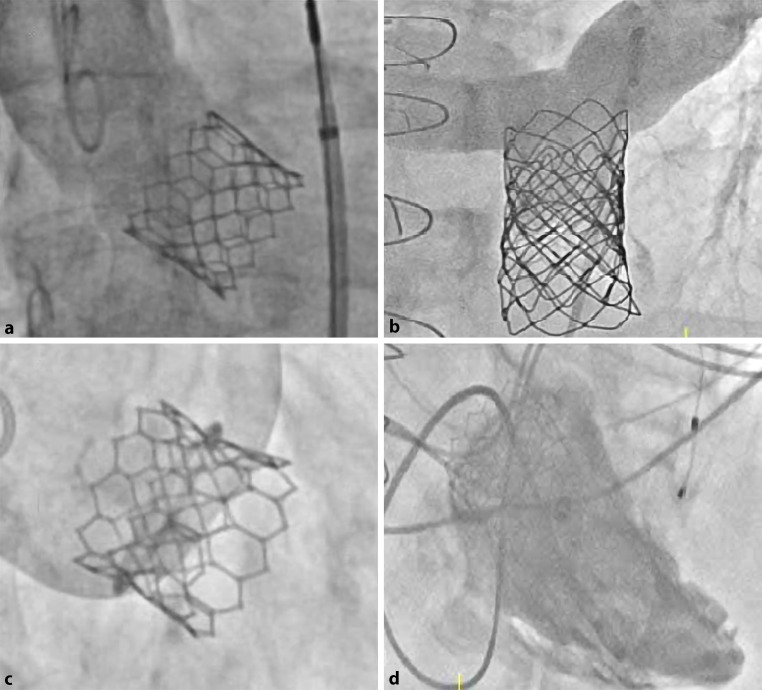
**a**, **b** The semilunar valves with an Edwards Sapien 3 (29 mm) in native aortic valve stenosis (*left panel*) and off-label use of an Edwards Sapien 3 (23 mm) after deployment of two overlapping stents in a pulmonary homograft (*right panel*). **c**, **d** The atrioventricular valves with off-label valve-in-ring, using Edwards Sapien 3 (26 mm) because of mitral valve insufficiency after mitral valve plasty (*left panel*), and off-label valve-in-ring, using Edwards Sapien 3 (23 mm) because of tricuspid valve insufficiency after tricuspid valve plasty (*right panel*)

We present four cases of successful percutaneous valve replacements, each in a different anatomical position. The percutaneous approach was the preferred treatment in these four patients due to extensive comorbidities. Approaches ranged from valve-in-native to valve-in-valve and valve-in-homograft for aortic, mitral and tricuspid, and pulmonary valve respectively (Fig. 1a–d). The Edwards Sapien 3 valve was used off-label in all cases except for the aortic valve replacement. Angiographic imaging showed no insufficiency in all valves.

Transcatheter aortic valve replacement has proven to be a good alternative treatment modality for surgical valve replacement [1]. Research on percutaneous valve implantation in pulmonic, mitral and tricuspid position is promising [2–4]. Developments in this field will be beneficial, particularly for patients who are unfavourable candidates for open-heart surgery. Future studies should focus on feasibility and, in particular, on the long-term outcome of percutaneous treatment of valvular heart disease.
